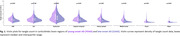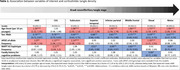# Tangle distribution is differentially influenced by age at onset, *APOE ε4*, and *MAPT H1* along the corticolimbic axis

**DOI:** 10.1002/alz.083978

**Published:** 2025-01-03

**Authors:** Baayla D. C. Boon

**Affiliations:** ^1^ Mayo Clinic, Jacksonville, FL USA

## Abstract

**Background:**

Alzheimer’s disease (AD) is heterogeneous in both its clinical and neuropathologic course. Age at onset and distribution of corticolimbic tangles can vary widely among individuals. Genetic risk factors *APOE ε4* and *MAPT H1* increase AD risk. However, the association with the spatial distribution of pathology remains a key gap in knowledge. We investigated the effects of age at onset, *APOE ε4*, *MAPT H1*, and family history of cognitive symptoms on corticolimbic tangle density variability to better distinguish how each factor contributes.

**Method:**

The FLorida Autopsied Multi‐Ethnic (FLAME) cohort was queried for neuropathologically confirmed AD cases (n = 1233). Tangle density (count per 0.125mm2) was quantified using thioflavin‐S staining in nucleus basalis of Meynert (nbM), hippocampal CA1 and subiculum, association cortices, and primary cortices. Cortical thickness was measured using digital pathology on hematoxylin‐eosin‐stained sections. We employed univariate and multivariate negative binomial regression models to evaluate the association of each factor on tangle density across the corticolimbic axis. Multivariate analysis was adjusted for significant variables from univariate analyses.

**Result:**

Donors with young‐onset (<65) AD (YOAD; n = 314) had more tangles than those with late‐onset AD (LOAD; n = 919) across most corticolimbic regions (**Fig. 1**). Except for CA1, younger age at onset was associated with a higher expected tangle density with an increase ranging from 8% in subiculum up to 99% in motor cortex (**Table 1**). Male sex was associated with lower expected tangle density throughout all corticolimbic regions. Being male associated the strongest in visual cortex with a 41% tangle density decrease. The *APOE ε4* allele associated with an increase of tangle density in the nbM, CA1, subiculum, and visual cortex, whereas the *MAPT H1* haplotype associated with a decrease in association and motor cortices. A positive family history for cognitive problems was associated with a 16% decrease of tangle density in visual cortex. Tangle density correlated closely with (sub)cortical thickness in the CA1+subiculum, superior temporal, inferior parietal, and middle frontal cortex (Spearman Rho = ‐0.39 ‐ ‐0.58, p<0.001).

**Conclusion:**

These data contribute to the concept that brain regions may experience aging and genetics differentially, underlining the need to distinguish these aspects when investigating AD disease mechanisms.